# Diverse Antibody Responses to Conserved Structural Motifs in *Plasmodium falciparum* Circumsporozoite Protein

**DOI:** 10.1016/j.jmb.2019.12.029

**Published:** 2020-02-14

**Authors:** Tossapol Pholcharee, David Oyen, Jonathan L. Torres, Yevel Flores-Garcia, Gregory M. Martin, Gonzalo E. González-Páez, Daniel Emerling, Wayne Volkmuth, Emily Locke, C. Richter King, Fidel Zavala, Andrew B. Ward, Ian A. Wilson

**Affiliations:** 1 Department of Integrative Structural and Computational Biology, The Scripps Research Institute, La Jolla, CA, 92037, USA; 2 Malaria Research Institute, Johns Hopkins Bloomberg School of Public Health, Baltimore, MD, 21204, USA; 3 Atreca Inc., Redwood City, CA, 94063, USA; 4 PATH's Malaria Vaccine Initiative, PATH Center for Vaccine Innovation and Access, Washington, DC, 20001, USA; 5 The Skaggs Institute for Chemical Biology, The Scripps Research Institute, La Jolla, CA, 92037, USA

**Keywords:** malaria, sporozoite, NANP repeats, type I β-turn, Trp-Asn interaction, Pf, *Plasmodium falciparum*, CSP, circumsporozoite protein, rsCSP, recombinant, shortened circumsporozoite protein, SPZ, sporozoite, mAb, monoclonal antibody, Fab, fragment of antigen binding, BSA, buried surface area, CDR, complementarity-determining region, ITC, isothermal titration calorimetry, BLI, biolayer interferometry, nsEM, negative-stain electron microscopy

## Abstract

Malaria vaccine candidate RTS,S/AS01 is based on the central and C-terminal regions of the circumsporozoite protein (CSP) of *P. falciparum*. mAb397 was isolated from a volunteer in an RTS,S/AS01 clinical trial, and it protects mice from infection by malaria sporozoites. However, mAb397 originates from the less commonly used *VH3-15* germline gene compared to the *VH3-30/33* antibodies generally elicited by RTS,S to the central NANP repeat region of CSP. The crystal structure of mAb397 with an NPNA_4_ peptide shows that the central NPNA forms a type I β-turn and is the main recognition motif. In most anti-NANP antibodies studied to date, a germline-encoded Trp is used to engage the Pro in NPNA β-turns, but here the Trp interacts with the first Asn. This “conserved” Trp, however, can arise from different germline genes and be located in the heavy or the light chain. Variation in the terminal ψ angles of the NPNA β-turns results in different dispositions of the subsequent NPNA and, hence, different stoichiometries and modes of antibody binding to rsCSP. Diverse protective antibodies against NANP repeats are therefore not limited to a single germline gene response or mode of binding.

## Highlights

• mAb397 from an RTS,S trial binds NANP repeats of malaria circumsporozoite protein.
• Protective mAb397 is encoded by the *VH3-15* gene that is rare for NANP antibodies.
• Anti-NANP mAbs bind type I β-turns in three modes using germline-encoded Trp residues.
• The Trp of mAb397 interacts with Asn instead of Pro in the NPNA type I β-turn.
• Diversity in NANP immune responses can aid in next-generation vaccine design.

## Graphical abstract

**Figure:**
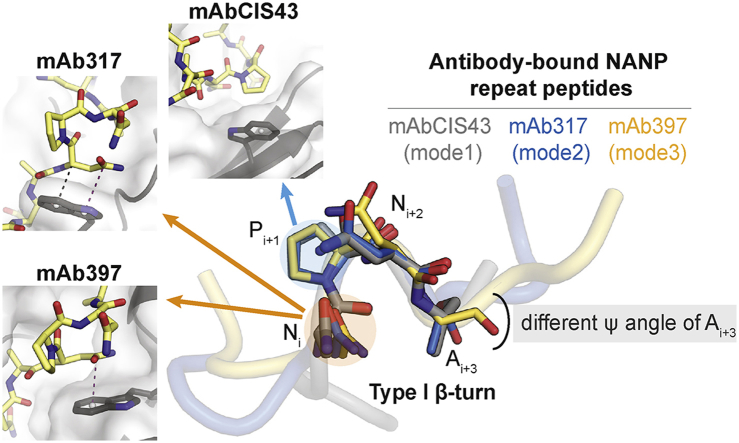

## Introduction

Malaria is a mosquito-borne disease caused by *Plasmodium* parasites with *P. falciparum* representing the most prevalent and deadly species infecting humans. Despite efforts to reduce mortality, malaria remains a world health threat with an estimated 219 million cases and 435,000 deaths in 2017, a majority of which are children under 5 years of age in sub-Saharan Africa [1]. The emergence of multidrug-resistant parasite strains and insecticide-resistant mosquitoes has slowed down the progress in malaria control and elimination and underlines the need for more sustainable measures to prevent malaria such as vaccines [[2], [3], [4]]. The pre-erythrocytic stage of *P. falciparum*, in which sporozoites from infected female mosquitoes are released into the human skin during a blood meal, is an ideal target for malaria vaccine development. Immunity at this stage would prevent the establishment of infection in the liver and thereby block parasite development and transmission [5].

*P. falciparum* (Pf) sporozoites are coated with the circumsporozoite protein (CSP), which is necessary for the development and migration of sporozoites in infected mosquitoes and for traversal, adhesion, and invasion of hepatocytes in humans [[6], [7], [8], [9]]. CSP is composed of an N-terminal domain containing a heparan sulfate binding site for hepatocyte adhesion [6], a central repeat region, and a structured C-terminal α-thrombospondin repeat (αTSR) [10] that is followed by a GPI anchor, which attaches CSP to the sporozoite membrane. The central repeat region of PfCSP is highly immunogenic [11], and in all *P. falciparum* strains with a CSP sequence available, the repeat region is composed of 1 NPDP repeat, 3–5 NVDP repeats, and 35–41 NANP repeats (e.g., a total of 1/4/38 of NPDP/NVDP/NANP motifs are present in the *P. falciparum* 3D7 strain) [[12], [13], [14], [15]]. The repeat region begins with the junctional NPDP sequence, typically followed by three alternations of NANP and NVDP sequences, and continues with the remaining NANP repeats, with most Pf strains having one NVDP interspersed in the middle of the long NANP repeat region. The most advanced malaria subunit vaccine is GSK's CSP-based RTS,S/AS01, which consists of 19 of the NANP repeats and the C-terminal αTSR linked to hepatitis B viral surface protein with AS01 as adjuvant [16]. Another vaccine candidate against *P. falciparum* is attenuated sporozoites, as in the Sanaria PfSPZ vaccine [17]. RTS,S/AS01 and Sanaria PfSPZ vaccines both elicit robust antibody responses against CSP [18,19]. However, antibody titers elicited and efficacy from both vaccines waned over time [[20], [21], [22]], emphasizing the need to improve current vaccines to induce more durable antibody protection with higher potency. Based on proven vaccine efficacy from the phase III clinical trial performed in young African children living in seven countries, a large-scale pilot implementation of the RTS,S vaccine, intended to reach approximately 1 million children, is being coordinated by the World Health Organization (WHO) in partnership with governments in Malawi, Ghana, and Kenya [23]. Other designs, including R21, another RTS,S-like vaccine, are undergoing early-stage testing in clinical trials [24,25].

Recently, structural and biophysical techniques have been applied to study the molecular basis of anti-NANP antibody recognition of CSP [26,27]. Such antibodies include those obtained from US volunteers vaccinated with RTS,S/AS01 in phase IIa clinical trials [28]; for example, mAb317 is one of the most protective antibodies identified to date along with mAb311 [29]. Other protective antibodies have been elicited in attenuated PfSPZ trials from either US, German, or African volunteers, such as mAbCIS43 [30], mAb1210 [31], mAb1450 [31], and mAbMGG4 [32], and from naturally infected patients in Africa, e.g., mAb663 [33]. Most of these antibodies originate from *VH3-33* or closely related *VH3-30* germline genes (mAb311, 317, MGG4, and 1210), or the *VH3-23* family (mAb580, 663, and 1450). Furthermore, mAbCIS43, mAbMGG4, and mAb311 not only bind NANP repeat peptides, but also recognize or cross-react with the junctional epitope, defined as the region between the N-terminal domain and the central NANP repeat region that contains the only NPDP sequence [30,32,34]. The epitopes of most of these antibodies have an NPNA motif exhibiting the type I β-turn (e.g., in mAb311, 317, 663, 1210, and CIS43) [[29], [30], [31],^33^,^34^], which is also observed in the crystal structure of free NANP peptide [35] and characterized by approximate φ and ψ angles of Pro (i+1) of −60° and −30°, and φ and ψ angles of Asn (i+2) −90° and 0°, respectively. An exception is the epitope of mAb1450, which has an extended conformation of the NANP repeats without any distinguishable β-turns [31]. These observations agree with the solution NMR results showing that free NANP peptides are in equilibrium between a disordered state and an ordered type I β-turn [36]. Another turn that is frequently observed among the NPNA motifs of anti-CSP antibodies is the Asn pseudo 3_10_ (e.g., in mAb311, 1210, and MGG4) [29,31,32,34]. The turn was first identified in the NANP repeat peptide bound to mAb311 [29,34] and was defined based on the similarity of the first Asn residue (i) found in the 3_10_-helix N-cap, namely: χ^1^ angle of −163° ± 20° and side-chain hydrogen bonding to the backbone NH of residue i+2 [37].

In this study, we investigated the structure and binding specificity of a protective monoclonal antibody mAb397, which was later derived from the MAL071 trial of RTS,S/AS01 vaccine [28]. In contrast to other published anti-NANP antibodies, mAb397 originates from the *VH3-15* gene, a less commonly observed *VH* germline gene in antibodies derived from naturally infected patients [33] and volunteers immunized with RTS,S [28] or PfSPZ [32]. Here we report the crystal structure and negative-stain electron microscopy (nsEM) reconstructions of mAb397 and compare the molecular basis of its binding to other published anti-NANP antibodies. Despite different germline gene usage, mAb397 has a similar binding mechanism to mAb317 through a germline-encoded Trp, which can be derived from either the *VH* or *VK* germline genes in different antibodies. The mAb397 epitope also contains an NPNA type I β-turn. When compared to the type I β-turns found in the epitopes of other anti-CSP antibodies, these turns can be classified into three modes, enabling the diversification of humoral immune responses. These structural insights may be utilized for design of next-generation CSP-based vaccines to aid in selection of protective types of antibodies from the immune repertoire.

## Results

### Evaluation of *in vivo* antibody protection and sporozoite binding

**Fig. 1:**
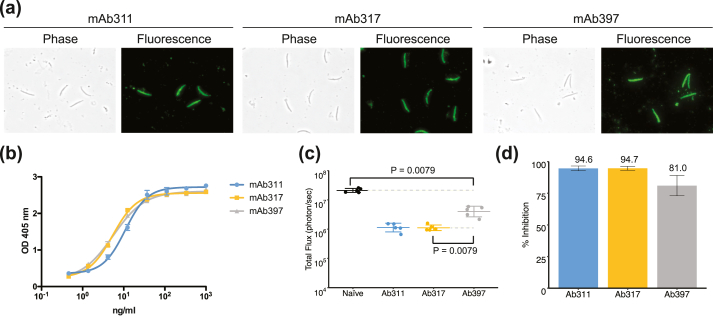
*In vivo* mice protection studies. (a) Representative mAb immunofluorescence reactivity (green) with Pf sporozoites. Phase-contrast and fluorescence channels are shown. (b) Binding of different concentrations of mAbs to PfSPZ in an ELISA. OD_405 nm_ = optical density at 405 nm. Error bars represent standard deviation (SD; *N* = 2). (c) Parasite liver burden load as measured by bioluminescence of luciferase-expressing transgenic *P. berghei* sporozoites after passive transfer of 100 μg of antibody in C57Bl/6 mice with mAb311 in blue, mA317 in orange, and mAb397 in gray. Data are represented as arithmetic mean ± 2SD (*N* = 5). Significant protection is observed for all antibodies compared with naïve mice (black; *p* = 0.0079; Mann–Whitney *U* test). (d) Inhibition of parasite development for mAb311 (blue bar, 94.6%), mAb317 (orange bar, 94.7%), and mAb397 (gray bar, 81.0%), respectively. The parasite liver burden load of mAb397 is significantly different from mAb311 and mAb317 (*p* = 0.0079; Mann–Whitney *U* test). Data are represented as geometric mean with error bars indicating standard deviation (*N* = 6).

Monoclonal antibody (mAb) 397 was later derived with other mAbs, including mAb311 and mAb317 [29,34], from the original MAL071 trial of RTS,S/AS01 vaccine [28]. The mAb397 is derived from germline gene *VH3-15* in comparison to mAbs 317 and 311 from the more common *VH3-33/VH3-30* family for anti-NANP antibodies to CSP (Fig. S1). mAb397 binds to *P. falciparum* sporozoites (PfSPZ), as determined by an immunofluorescence assay, in a similar way to mAb311 and mAb317 (Fig. 1a). All three mAbs showed dose-dependent binding to PfSPZ in an ELISA with the maximal effective concentration around 100 ng/ml, similar to that observed for mAb311 and mAb317 (Fig. 1b). To measure reduction of parasite liver burden load, mice (*N* = 5) were administered mAb 397, 311, or 317 at a dose of 100 μg by intravenous injection (IV) and then challenged after 16 h with chimeric sporozoites (*P. berghei* sporozoites expressing full-length *P. falciparum* CSP) [38]. mAb397 showed ~fivefold reduction (81% inhibition) of liver burden load as compared to naïve infected mice, whereas mAb311 and mAb317 exhibited a ~19-fold reduction (95% inhibition) at the same dose (Fig. 1c and d). These results indicate that mAb397 is less effective at 100 μg/mouse in this liver burden assay compared to previously reported antibodies mAb311 and mAb317 [29] (Fig. 1c). However, mAb397 conferred 100% protection at 300 μg, similar to mAb311, as assessed by blood parasitemia in mice (*N* = 6) when mAbs were passively transferred by IV, and the mice were then subjected to subsequent mosquito bite challenge (Table S1).

### Crystal structure of Fab397 in complex with NPNA_4_ peptide

**Fig. 2:**
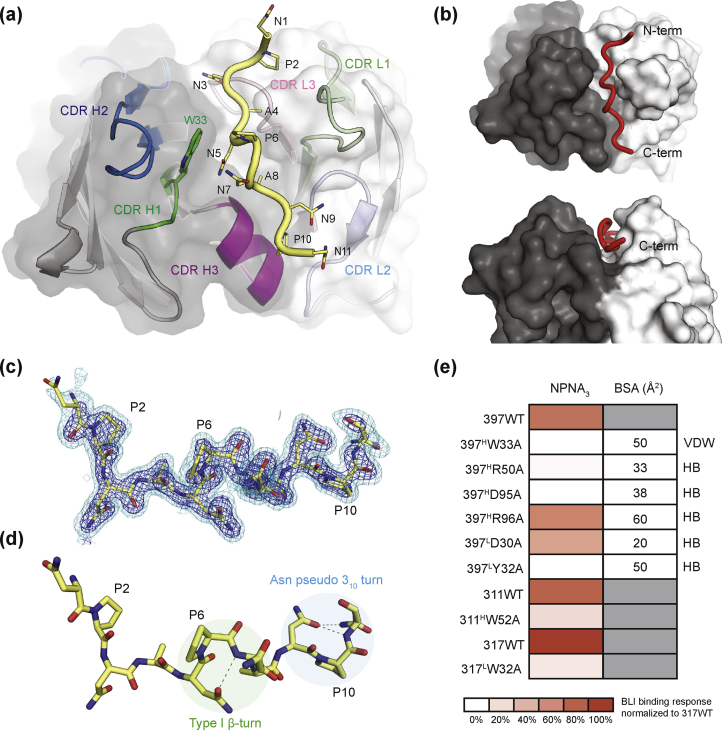
Structural analysis of Fab397-NPNA_4_ peptide complex. (a) Crystal structure of Fab397-NPNA_4_ peptide complex. The paratope on the Fab397 variable domains is shown in a cartoon representation overlaid with a transparent surface representation. The complementarity-determining regions (CDRs) are colored green (H1), blue (H2), magenta (H3), light green (L1), light blue (L2), and pink (L3). Gray and white surfaces represent the heavy and light chain, respectively. The NPNA_4_ peptide (yellow) is shown in backbone tube representation with side chains as sticks. All residues of the peptide are labeled, and the germline-encoded ^H^Trp^33^ side chain is highlighted in green sticks. (b) Two views of the binding groove of Fab397 with heavy (black) and light (white) chains in a solid surface and NPNA_4_ peptide in a tube representation (red). (c) The conformation of the NPNA_4_ peptide (yellow carbons); only residues 1–11 of the peptide have interpretable electron density. The modeled peptide is shown in a 2Fo-Fc electron density map contoured at 2.0σ (blue) and 0.8σ (cyan). (d) The type I β-turn (green circle) and Asn pseudo 3_10_ turn (blue circle) are highlighted together with connecting hydrogen bonds (dashes). (e) Binding response from biolayer interferometry (BLI) of Fabs 397, 311, and 317 wild-type and mutants to NPNA_3_ peptide. The responses are normalized to Fab317 WT. Buried surface areas (BSA) of Fab397 residues mutated to Ala are indicated in Å^2^. The interactions of mutated residues to the repeat peptide observed in the Fab397-NPNA_4_ crystal structure are indicated as VDW for van der Waals interaction and HB for hydrogen bonds.

The Fab397-NPNA_4_ peptide complex was crystallized and X-ray diffraction data collected to 1.75 Å resolution in space group P2_1_2_1_2_1_, with one Fab-peptide in the asymmetric unit (Table 1). Six substitutions and one deletion (from ^112^SSASTKG^118^ to ^112^VSRRLP^117^) were introduced into the flexible elbow region of the Fab397 heavy chain to stabilize the Fab through a more ordered constant region and hence facilitate Fab crystallization and higher resolution structure determination as previously described [39]. The electron density of the NPNA_4_ peptide was well-defined for 11 out of 16 residues for ^1^NPNANPNANPN^11^ (i.e., Asn^1^ to Asn^11^) (Fig. 2a–d and Fig. S2). The absence of electron density after Asn^11^ is likely due to its flexibility after exiting the Fab binding groove (Fig. 2a and b). The first N-terminal NPNA repeat in the binding pocket does not form a defined secondary structural motif. However, dihedral angle analysis indicates that the second repeat located in the center of the antibody binding site adopts a type I β-turn, which is stabilized by a hydrogen bond between the side chain of Asn^5^ (i) and the backbone of Asn^7^ (i+2) (Fig. 2d). The third repeat forms an Asn pseudo 3_10_ turn, which is stabilized by a hydrogen bond from the side chain of Asn^9^ (i) to both the main chain and side chain of Asn^11^ (i+2) (Fig. 2d). The peptide resides in a groove between the heavy and light chains of Fab397 (Fig. 2a and b). All complementarity-determining region (CDR) loops contribute to the binding groove with the heavy and light chains providing roughly an equal proportion of the total buried surface area (BSA; 592 Å^2^ on the Fab). The heavy chain contributes 44.5% of BSA from CDR H1 (8.4%), H2 (10.6%), and H3 (25.5%), whereas the light chain contributes 55.5% of BSA from CDR L1 (25.6%), L2 (6.8%), L3 (18.3%), and ^L^Tyr^54^ from the framework region (4.8%).

**Table 1:** X-ray data collection and refinement statistics for Fab397 with NPNA_4_

Data collection	Fab397-NPNA_4_
Beamline	APS23-IDB
Wavelength (Å)	1.03317
Space group	P2_1_2_1_2_1_
Unit cell parameters (Å, °)	a = 61.27, b = 77.49, c = 87.70
	α = β = γ = 90
Resolution (Å)	50.00–1.75 (1.78–1.75)^a^
Unique reflections	41,579 (1315)^a^
Redundancy	10.6 (3.9)^a^
Completeness (%)	95.3 (61.1)^a^
<I/σ_I_>	24.5 (1.2)^a^
R_sym_^b^ (%)	10.4 (76.0)^a^
R_pim_^b^ (%)	3.1 (33.9)^a^
CC_1/2_^c^ (%)	96.9 (82.3)^a^
Refinement statistics	
Resolution (Å)	35.66–1.75
Reflections (work)	41,411
Reflections (test)	2105
R_cryst_^d^/R_free_^e^ (%)	19.0/22.5
No. of atoms	
Fab	6335
Peptide	144
Water	253
Average B-value (Å^2^)	
Fab	35
Peptide	35
Water	37
Wilson B-value (Å^2^)	24
RMSD from ideal geometry	
Bond length (Å)	0.004
Bond angle (°)	0.65
Ramachandran statistics^f^	
Favored (%)	98.17
Outliers (%)	0.00

a Numbers in parentheses refer to the highest-resolution shell.

b *R*_sym_ = Σ_*hkl*_ Σ_*i*_ | I_*hkl,i*_ - <I_*hkl*_> |/Σ_*hkl*_ Σ_*i*_ I_*hkl,i*_ and R_*pim*_ = Σ_*hkl*_ (1/(n-1))^1/2^ Σ_*i*_ | I_*hkl,i*_ - <I_*hkl*_> |/Σ_*hkl*_ Σ_*i*_ I_*hkl,i*_, where I_*hkl,i*_ is the scaled intensity of the i^th^ measurement of reflection h, k, l, <I_*hkl*_> is the average intensity for that reflection, and *n* is the redundancy.

c CC_1/2_ = Pearson correlation coefficient between two random half datasets.

d *R*_cryst_ = Σ_*hkl*_ | *F*_o_ - *F*_c_ |/Σ_*hkl*_ | *F*_o_ |x 100, where *F*_o_ and *F*_c_ are the observed and calculated structure factors, respectively.

e *R*_free_ was calculated as for *R*_cryst_, but on a test set comprising 5% of the data excluded from refinement.

f From MolProbity [55].

**Fig. 3:**
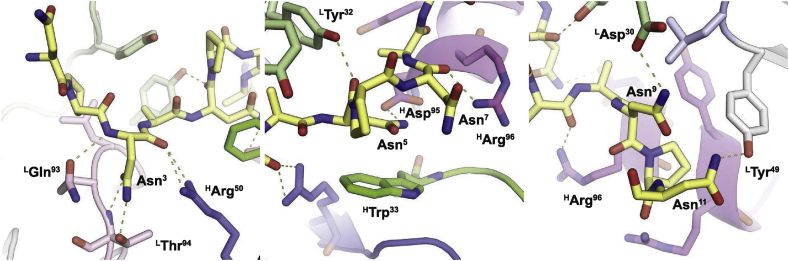
Hydrogen bonds between Fab397 and NPNA_4_ peptide. The three panels from left to right indicate interactions from the N- to C-terminus of the peptide (yellow carbons) with the Fab. Hydrogen bonds are shown with olive dashes. Amino acids are shown with a superscript indicating residue number in the peptide and Fab (Kabat numbering). The Fab residues have either H or L superscript to indicate heavy and light chain, respectively, and are colored based on the complementarity-determining regions (CDRs): green (H1), blue (H2), magenta (H3), light green (L1), light blue (L2), and pink (L3).

Most of the hydrogen bonds formed between Fab397 and peptide are with the N-terminal half of the peptide (Fig. 3 and Table S2). Asn^3^ and Asn^5^ display extensive interactions, as indicated by their large BSAs (73 and 70 Å^2^, respectively). Both side chain and main chain of Asn^3^ engage in a hydrogen bonding network with ^L^Gln^93^, ^L^Thr^94^, and ^H^Arg^50^, whereas Asn^5^ hydrogen bonds with ^L^Tyr^32^ and ^H^Asp^95^ (Fig. 3 and Table S2). The Asn^5^ conformation is further stabilized by a π–π interaction with ^H^Trp^33^ (Fig. 2, Fig. 3, Fig. 7; see also Methods), which is the only CDR H1 residue that contributes to the BSA. In contrast, the C-terminal half of the peptide only has three hydrogen bonds between Asn^7^ and ^H^Arg^96^, Asn^9^ and ^L^Asp^30^, and Asn^11^ and ^L^Tyr^49^ (Fig. 3 and Table S2). However, the contributions from Ala^8^ (57 Å^2^) and Pro^10^ (68 Å^2^) on the peptide indicate that the C-terminal half of the peptide is engaged in substantial van der Waal interactions with CDR H3 (151 Å^2^).

### Affinity and mutational studies of Fab397 with NANP peptides

To determine the binding affinity of Fab397 to the NANP repeat peptide, isothermal titration calorimetry (ITC) was performed for Fab397 with NPNA_2_, NPNA_4_, and NPNA_8_ peptides. Fab397 binds NPNA_2_ with low affinity (*K*_*d*_ = 3.5 ± 0.3 μM) (Table 2 and Fig. S3). However, the affinity increases substantially with the NPNA_4_ peptide (*K*_*d*_ = 44 ± 3 nM), which suggests that the weaker binding of Fab397 to the NPNA_2_ peptide is due to it being shorter than the minimal epitope defined in our crystal structure (Fig. 2). Fab397 also binds to the NPNA_8_ peptide with a similar affinity (*K*_*d*_ = 41 ± 3 nM) as NPNA_4_ (Table 2 and Fig. S3). The binding of Fab397 to NPNA_4_ is comparable to that of Fab317 with NPNA_3_ (*K*_*d*_ = 78 ± 16 nM) [29]. The N-values derived from ITC with the NPNA_2_ and NPNA_4_ peptides (1.25 ± 0.02 and 1.14 ± 0.01, respectively) indicate a peptide:Fab binding ratio of 1:1, whereas that of NPNA_8_ is 0.56 ± 0.01, which suggests that two Fabs bind per peptide. These N-values also support three NPNA repeats being the minimal binding epitope of Fab397 for binding to free peptides, as observed in the crystal structure (Fig. 2).

**Table 2:** Dissociation constants of Fab397 for (NANP)_2_, (NANP)_4_, and (NANP)_8_ peptides from ITC affinity measurements

	No. of sites	*K*_d_ (μM)	ΔH (cal/mol)	ΔS (cal^−1^·mol^−1^·K^−1^)	TΔS (cal·mol^−1^)
(NANP)_2_	1.25 ± 0.02	3.5 ± 0.3	−13303 ± 556	−19.7 ± 2.0	−5859 ± 593
(NANP)_4_	1.14 ± 0.01	0.044 ± 0.003	−19915 ± 102	−33.2 ± 0.5	−9884 ± 147
(NANP)_8_	0.56 ± 0.01	0.041 ± 0.003	−40090 ± 143	−100.7 ± 0.6	−30024 ± 179

Data are represented as the arithmetic mean ± standard deviation of quadruplet experiments.

Alanine substitution mutations were introduced into Fab397 to assess the contribution of particular residues in the Fab binding groove to NPNA-repeat peptide binding. Binding of the mutated Fabs to biotinylated NPNA_3_ peptide was measured using biolayer interferometry (BLI). Mutations were chosen based on residues with relatively large BSA contributions to the Fab–peptide interaction. Specifically, residues interacting with the type I β-turn in the NANP repeat peptide (^H^Trp^33^, ^H^Asp^95^, and ^L^Tyr^32^) and additional residues (^L^Asp^30^, ^H^Arg^50^, and ^H^Arg^96^) were mutated to 397^H^W33A, 397^H^D95A, 397^L^Y32A, 397^L^D30A, 397^H^R50A, and 397^H^R96A, respectively (Fig. 2, Fig. 3 and Fig. S4). Furthermore, we mutated ^H^Trp^52^ in Fab311 and ^L^Trp^3^^2^ in Fab317 (Fab311^H^W52A and Fab317^L^W32A, respectively; see also Fig. 7) to assess the importance of a tryptophan in the binding site of anti-NANP antibodies from the *VH3-33* and *VH3-30* genes since ^H^Trp^52^ was shown to play a critical role in binding of mAbMGU10 (*VH3-33*) to the junctional peptide [32]. All binding responses from BLI were normalized to that of 317 WT (binding response = 100%), which had the highest response among this panel of antibodies. Alanine substitution of Fab397 residues that interact with the peptide type I β-turn completely abrogated binding (^H^W33A, ^H^D95A, and ^L^Y32A) (Fig. 2, Fig. 3 and Fig. S4). Very weak binding was observed for ^H^R50A (9%), whereas ^L^D30A and ^H^R96A only slightly affected binding with a relative response of 66% and 77%, respectively (Fig. 2, Fig. 3 and Fig. S4). In addition, Fab311 ^H^W52A and Fab317 ^L^W32A mutations both substantially disrupted NPNA_3_ binding (26% and 20% relative binding; Fig. 2e and Fig. S4).

### Negative-stain electron microscopy of Fab397 with NPNA_8_ peptide and a recombinant shortened CSP construct

**Fig. 4:**
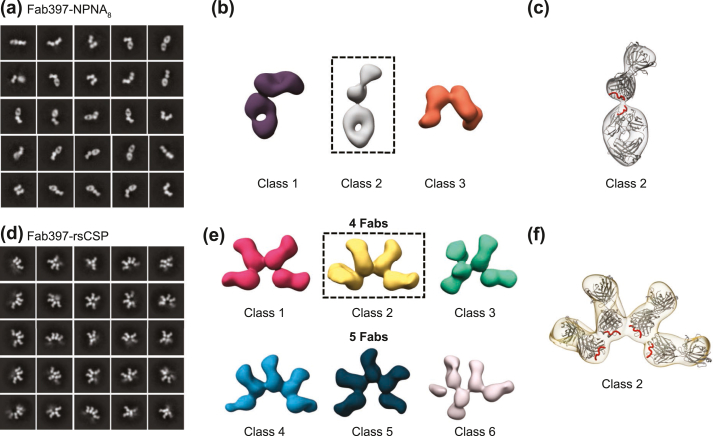
Fab397 binding to NPNA-repeat peptides and rsCSP by negative-stain EM. (a) Representative class averages from nsEM for Fab397-NPNA_8_. (b) Refined classes of Fab397-NPNA_8_. (c) Crystal structure of Fab397-NPNA_8_ was docked into one of the refined EM maps. (d) Representative class averages for Fab397-rsCSP and (e) refined classes of Fab397-rsCSP, consisting of either four or five Fabs bound. (f) The crystal structure of Fab397 in complex with the NANP repeat peptide was docked into the refined EM map. The Fab397 crystal structure is colored in light gray with the peptide as a red tube.

The binding stoichiometry and molecular organization of Fab397 to CSP were approximated using the NPNA_8_ peptide and a recombinant shortened construct (rsCSP), which has an NPDP/NVDP/NANP repeat ratio of 1/3/19 instead of 1/4/38 for the *P. falciparum* 3D7 strain. The rsCSP has been shown to act as a good substitute for antibody binding to full-length CSP [34]; however, whether it fully represents binding to intact CSP on the surface of sporozoites still remains to be determined. The 2D class averages of the Fab397-NPNA_8_ complex show that two Fab molecules bind to the peptide (Fig. 4a–c and Figs. S5-6); such a binding stoichiometry corresponds to the minimal binding epitope (NPNANPNANPA) defined from the crystal structure (Fig. 2). In addition, visual inspection of the class averages for the Fab397-rsCSP complex shows an average binding stoichiometry of 4–5 Fabs per rsCSP molecule (Fig. 4d–e and Fig. S6. The crescent organization and tight packing of Fab397 molecules around rsCSP suggest that they could contain inter-Fab contacts (Fig. 4f), similar to those observed for Fab311 and Fab1210 in previous studies [31,34]. Such inter-Fab contacts facilitated simultaneous binding of 11 copies of Fab311 to the spiral conformation of rsCSP, with an angular twist between the Fab variable domains of ~77° [34]. In Fab1210, two molecules adopt a head-to-head configuration and display inter-Fab contacts with a 133° angle between the two Fabs, when bound to an NANP repeat peptide [31]. For Fab397, the docking model of the Fab crystal structure into the nsEM reconstruction of Fab397-rsCSP complexes shows angles that appear to vary from 30° to 60° (Fig. S6). The average distance between the last Asn C_α_ of the peptide (NPNANPNANPN) in one Fab and the first Asn C_α_ in the peptide of the adjacent Fab from the docking model of the Fab397-rsCSP complex (Fig. 4f) is 18.7 ± 2.6 Å, which translates to about 5 amino acids apart (using an average C_α_–C_α_ distance of 3.8 Å in the Fab397-bound peptide). Thus, it is possible that each Fab397 epitope may be separated by 5 amino acids (i.e., NPNANPNANPN**ANPNA**NPNANPNANPN, where the underlined residues are the epitope in the Fab397 crystal structure, and the residues in bold are the proposed linker sequence). This spacing would differ from Fab311 where the epitope is exactly two NPNA repeats with no linking residues [34]. However, the nsEM class averages for the Fab397-NPNA_8_ complex exhibit great variability in the angles between the two Fabs (80° in class 1, 140° in class 2, and 60° in class 3; Fig. 4a, b and Fig. S6), which indicates flexibility of two Fabs bound to the peptide. In addition, the conformational heterogeneity of the Fab397-rsCSP hindered the processing of the complexes to high resolution with cryo-EM (data not shown). These results suggest that the two Fabs may not be stabilized by inter-Fab homotypic contacts as observed in two previous complexes [31,34]. Such a conclusion is supported by the ITC results, since inter-Fab contacts should lead to an increase in avidity in Fab binding to peptide due to cooperativity [31]. However, the binding affinity of Fab397 to the NPNA_8_ peptide is identical to the NPNA_4_ peptide, even though the N-value indicates that two Fabs are bound per NPNA_8_ peptide (Table 2 and Fig. S3).

### Dihedral angle analysis of type I β-turns and Asn pseudo 3_10_ turns in antibody-bound peptides

**Fig. 5:**
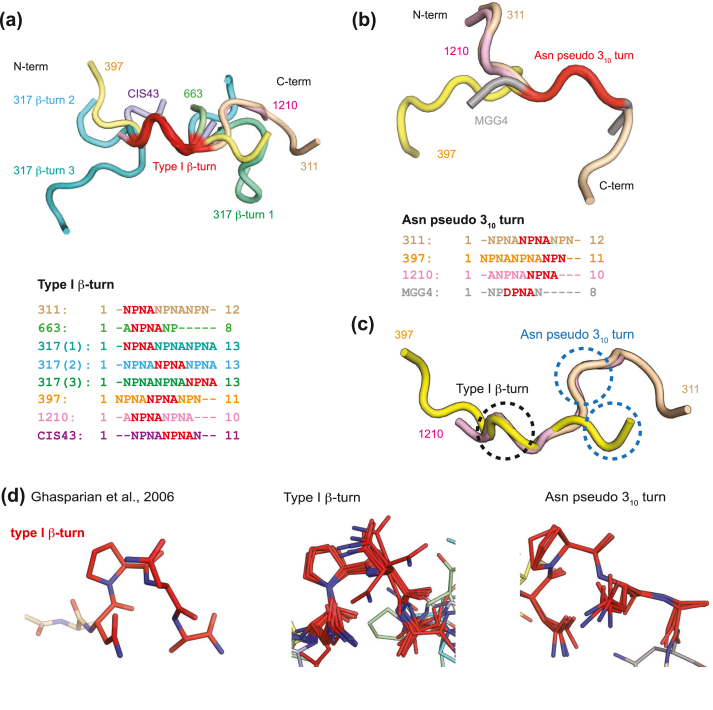
Comparison of the type I β-turns and Asn pseudo 3_10_ turns from peptide complexes with anti-NANP antibodies. (a–b) Cartoon tube representation of NANP repeat peptides (different colors) aligned based on (a) type I β-turn and (b) Asn pseudo 3_10_ turn, contained within the peptide and colored in red. Sequence alignment of NANP peptides visible in the respective structures is shown below with residues involved in (a) type I β-turn and (b) Asn pseudo 3_10_ turn highlighted in red. (c) Fab311-, 1210-, and 397-bound peptides were aligned based on their type I β-turn (circled in black). The Asn pseudo 3_10_ turns present in these peptides are highlighted with blue circles. (d) Stick representation of type I β-turn (red) in the NANPNA crystal structure from Ghasparian et al. [35], and the NPNA type I β-turn and Asn pseudo 3_10_ from peptides aligned in (a) and (b), respectively.

**Fig. 6:**
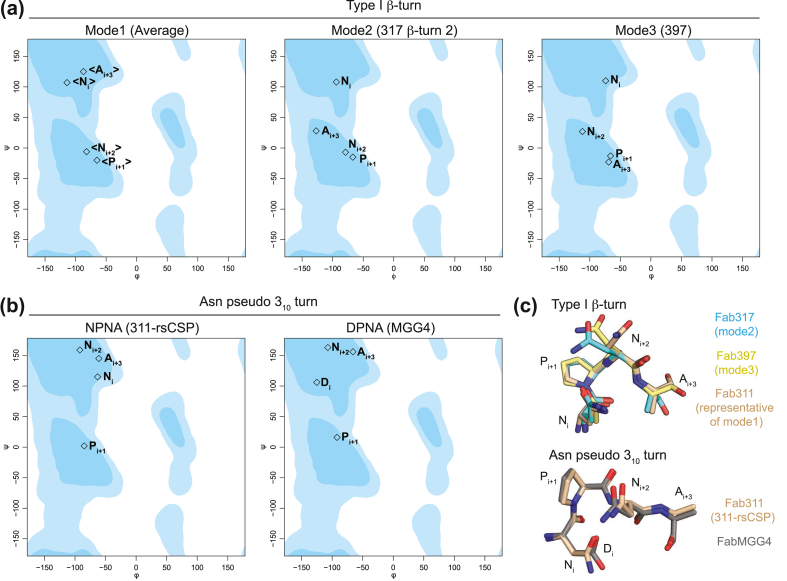
Ramachandran plots of dihedral angles of the type I β-turn and Asn pseudo 3_10_ turn adopted by the NPNA motif. The NPNA motif is labeled as N_i_, P_i+1_, N_i+2_, and A_i+3_ respectively. (a) The type I β-turns are classified into three modes based on the ψ angle of A_i+3_. Antibodies with the type I β-turn that represents each mode are indicated (see also Table S3). Dihedral angles labeled in angle brackets < > are the average values from different mAbs. (b) Dihedral angles of the Asn pseudo 3_10_ turn from the peptide in the Fab311-rsCSP complex [34] and in FabMGG4 [32]. (c) Stick representations of the three modes of type I β-turn (Fab311-bound peptide represents mode 1; see also Table S3), and the Asn pseudo 3_10_ turn with dihedral angles plotted in panel (b).

All previously published anti-CSP antibody structures with bound peptides that form an NPNA type I β-turn or Asn pseudo 3_10_ turn were analyzed to compare the similarities and differences of their peptide conformations with that of Fab397. An NPNA type I β-turn is present in six Fab-peptide complexes for Fabs 311, 317, 397, 663, 1210, and CIS43, with Fab317 having three type I β-turns [[29], [30], [31],^33^] (Fig. 5, Fig. S7 and Table S3; the Fab317 turns are labeled 1–3). On the other hand, only four Fabs (311, 397, 1210, and MGG4) have peptides with an Asn pseudo 3_10_ turn [29,31,32]; in the MGG4–peptide complex, the pseudo 3_10_ turn occurs in the DPNA motif [32], which is structurally related to the more common NPNA motif (Fig. 5, Fig. S7 and Table S3). For antibody-bound peptides that exhibit a type I β-turn, we found that dihedral angles of the NPNA turn motifs are quite similar for N_i_, P_i+1_, and N_i+2_, but differ in the terminal A_i+3_ residue (Fig. 6a). Notably, the type I β-turn from these NPNA-repeat peptides can be classified into three modes based on the dihedral angles of A_i+3_ (Fig. 6a and Table S3). Mode 1 contains the type I β-turn in peptides bound to Fabs 311, 1210, 663, CIS43, and the first and third type I β-turn from the Fab317-bound peptide (Fig. 6a, Fig. S7 and Table S3). These β-turns have positive ψ angles for A_i+3_ with an average of 125 ± 29° in the β-region of the Ramachandran plot. On the other hand, mode 2, with a slightly positive A_i+3_ ψ angle of 28° between the β and α regions, is only present in Fab317-bound peptide, which exhibits an unusual conformation of three successive type I β-turns [29] (mode 1, mode 2, mode 1) from N-to-C termini (Fig. 6a, Fig. S7 and Table S3). Interestingly, our analysis indicates that only the Fab397-bound peptide exhibits a type I β-turn with a negative A_i+3_ ψ angle of −23° in the helical region (Fig. 6a and Table S3). Such different A_i+3_ ψ angles could therefore restrict the orientation and relative disposition of the following NPNA repeat (Fig. 5, Fig. 6c, as shown from the carbonyl group of A_i+3_). For the Asn pseudo 3_10_ turn, only Fab311 and FabMGG4 have a complete NPNA motif where the terminal A_i+3_ dihedral angles can be examined. We found that the dihedral angles of all residues in the Asn pseudo 3_10_ turn from both Fab311 and MGG4 peptides are similar, except for replacement of N_i_ by D_i_, which is part of the junctional peptide sequence (Fig. 6b, Fig. S7 and Table S3). The terminal A_i+3_ from the Asn pseudo 3_10_ turns of both Fab311 and FabMGG4 bound-peptide have ψ angles of 145° and 156°, respectively, which is similar to the distribution of mode 1 A_i+3_ ψ angles from type I β-turns (Fig. 6a and b).

## Discussion

Most antibodies studied to date recognize the NANP repeat region in CSP and have been obtained from RTS,S/AS01 vaccination (mAb311, mAb317, and mAb397) [29], naturally infected patients (mAb663) [33], and volunteers immunized with the Sanaria PfSPZ vaccine (mAb1210, mAb1450, and mAbCIS43) [30,31]. In addition to pure NANP binding, some of these antibodies, such as CIS43 and MGG4, also bind to repeat regions containing the junctional NPDP and NVDP sequences [30,32]. Here, we show that human mAb397 obtained from the RTS,S/AS01 phase IIa clinical trial binds the NANP repeat region with high affinity and confers sterile protection against transgenic *P. berghei* parasites expressing *P. falciparum* CSP *in vivo* at a dose of 300 μg/mouse (Table S1). In the crystal structure, the Fab397-bound peptide adopts a type I β-turn (^5^NPNA^8^) followed by an Asn pseudo 3_10_ turn (^9^NPN^11^) (Fig. 2a–d). This type I β-turn in NANP-repeat peptides, which had previously been observed in free peptides by solution NMR [36] and in crystal structures [35], is consistently present in most anti-NANP-repeat antibodies, such as Fab311, Fab317, Fab663, Fab1210, and FabCIS43 (Fig. 5 and Table S3) [[29], [30], [31],^33^]. On the other hand, the pseudo Asn 3_10_ turn was first observed in the Fab311-bound NANP peptide and in the junctional peptide in mAbMGG4 [29,32,34].

### The role of Trp in antibody recognition of NANP repeats

**Fig. 7:**
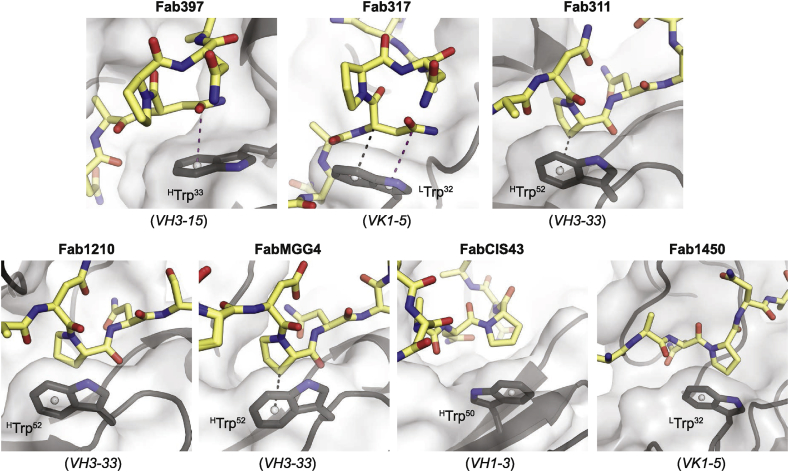
Interactions between germline-encoded Trp residues in anti-CSP antibodies and Pro or Asn residues in NANP repeat peptides. The peptides (yellow carbons) and Trp residues (dark gray carbons) are represented as sticks with the rest of the Fab shown in cartoon representation. Light gray, transparent surfaces of the Fabs are also displayed. CH–π interactions as defined previously [61] (see also Table 3) are indicated as black dash lines, whereas the π–π interactions were determined based on the criteria from previous study [62] and are shown as a magenta dash line. Pseudoatoms representing the center of the Trp 5- or 6-membered rings are shown as spheres. Top left to right, Fab: 397, 317 [29], 311 [29]. Bottom left to right, Fab: 1210 [31], and MGG4 [32], CIS43 [30], and 1450 [31]. The germline genes that encode these Trp residues are indicated.

The type I β-turns and Asn pseudo 3_10_ turns represent key structural features in the NANP-repeat region of PfCSP, with the immune system consistently using a germline-encoded Trp residue to recognize these turns (Fig. 7). The important contribution of this Trp residue in PfCSP binding was first highlighted by ^H^Trp^52^ of mAb MGU10, a junctional-region-binding antibody encoded by the *VH3-33* gene. Serine substitution of this residue in MGU10 resulted in a complete loss of binding to the junctional peptide [32]. For anti-CSP antibodies whose structures have been published, we identified seven antibodies that utilize a germline-encoded Trp to interact with the NANP peptide. The Trp in these antibodies engage either Pro or Asn in the peptide with different interactions. ^H^Trp^33^ from the *VH3-15* gene in Fab397 forms a π–π interaction with the side-chain amide of Asn in the type I β-turn (Fig. 7). A similar π–π interaction is also observed for ^L^Trp^32^ encoded by the *VK1-5* gene in Fab317 [29]. In contrast, CH–π and van der Waals interactions are observed between germline-encoded Trp residues and NANP repeat peptide Pro residues (Fig. 7 and Table 3). For example, *VH3-33*-encoded ^H^Trp^52^ interacts with the Pro residue in the Asn pseudo 3_10_ turn in the peptide bound to Fab311, 1210, and MGG4 [29,31,32,34] (Fig. 7 and Table 3). On the other hand, *VH1-3*-encoded ^H^Trp^50^ interacts with the type I β-turn Pro in the NANP peptide bound to CIS43 [30] (Fig. 7). The *VK1-5*-encoded ^L^Trp^32^ (same as in Fab317) was also observed to interact with Pro in the NANP peptide bound to Fab1450, although it is part of an extended NANP repeat conformation [31]. These Trp residues play a crucial role in peptide recognition as binding studies show that serine/alanine substitution in ^H^Trp^52^ from MGU10 [32], ^H^Trp^33^ from Fab397, ^L^Trp^32^ from Fab317, and ^H^Trp^52^ from Fab311 substantially disrupts the binding of the antibodies to repeat peptides (Fig. 2e).

**Table 3:**
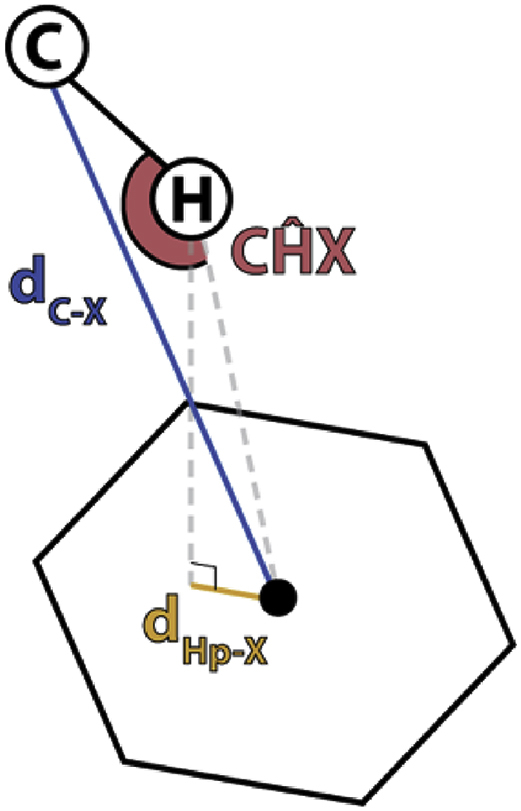
CH–π interaction of Pro or Asn residues in NANP peptides with Fabs. The Pro and Asn interact with the six-membered ring of the germline-encoded Trp residue in anti-CSP antibodies as shown in Fig. 7.

Antibody	CHX angle	d_C-X_	d_Hp-X_	Donor	Type
311	135°	3.6 Å	0.68 Å	Pro-C_β_	C_ali_-Aro
317	131°	3.5 Å	0.66 Å	Asn-C_α_	C_α_-Aro
MGG4	147°	3.9 Å	0.58 Å	Pro-C_β_	C_ali_-Aro

CH–π interactions are determined based on the criteria from Brandl et al. [59]. The angle between the carbon atom, hydrogen atom, and center of the Trp six-membered ring (CĤX) must be greater than 120°. The distance from the carbon atom to the center of the Trp six-membered ring (d_C-X_) must be less than 4.5 Å. Lastly, the distance of the hydrogen atom projected onto the π-plane and the center of mass of the π-plane (d_Hp-X_) must be less than 1.2 Å. C_ali_ (aliphatic carbon) is defined as the side-chain carbon of the interacting amino acid.

In fact, surveys of antibody structures have shown that aromatic residues, such as Trp and Tyr, are prevalent in antibody paratopes [[40], [41], [42], [43], [44]]. Aromatic side chains are considered to be favorable due to their abilities to facilitate antigen recognition through a collection of noncovalent interactions e.g., π–π, cation–π, anion–π, or CH–π interactions [[45], [46], [47]], which correspond to our findings in anti-NANP antibodies. The observations in this study emphasize that the human humoral immune system is well equipped to recognize *P. falciparum* sporozoites through a diverse repertoire of antibodies that contain, in this case, a *single* well-positioned Trp that interacts with the NANP peptide using either Pro or Asn, which are mostly part of type I β-turns or Asn pseudo 3_10_ turns. Since these Trp residues described so far only originate from the germline V region, our analysis supports previous observations that naïve human B-cells are predisposed for recognition of the NANP repeats in CSP independently of somatic hypermutations [33]. Additionally, our results agree with the speculation that the spatial geometries required for interactions, such as π–π or CH–π, involving aromatic side chains, could contribute to the specificity of antibody–antigen recognition [46]. The role that Trp side chains can play in antibody–antigen recognition was also demonstrated in the design of synthetic antibody, Fab37, against a receptor tyrosine kinase HER2 with a paratope composed mostly of Trp and Ser derived from binary phage display library [48]. In this antibody, Trp residues establish direct contacts to the antigen, where Ser residues allow for conformational diversity in the CDRs. However here, only a single germline-encoded Trp helps confer specificity of antibodies to the type I β-turns or the Asn pseudo 3_10_ turns in the PfCSP repeat region through either CH–π interaction with Pro or π–π interaction with Asn. Future characterizations of anti-NANP antibodies will help verify this notion and/or reveal the contribution of alternative aromatic residues in antibody-NANP-repeat recognition.

### Structural diversity of the NPNA type I β-turns

In addition, the dihedral angle analysis shows three different conformations of the terminal Ala residue in NPNA type I β-turns (A_i+3_), which influence the direction and disposition of the successive NPNA motif (Fig. 5, Fig. 6). Turns with a positive A_i+3_ ψ angle (125 ± 29°) are more common (Fig. 6a, Fig S7, and Table S3) and represent the first mode that could restrict the relative disposition of the next NPNA repeat. The importance of this conformation is shown in the cryo-EM structure of Fab311 in complex with rsCSP where type I β-turns and Asn pseudo 3_10_ turns repeatedly interchange each with identical mode 1 conformations for the terminal A_i+3_ residues [34]. The same repeated peptide conformation is also observed in the structure of Fab1210 in complex with an NANP_5_ peptide [31]. The second mode that influences the relative disposition of successive NPNA repeats is observed in the second type I β-turn of the Fab317 epitope and characterized by a small positive A_i+3_ ψ angle of 28° (Fig. 5, Fig. 6a and Table S3). Lastly, the third mode is characterized by a distinct negative A_i+3_ ψ angle (−23°) and is found in the type I β-turn of the Fab397 epitope (Fig. 6a, Fig S7, and Table S3).

Overall, our data demonstrate that anti-CSP antibodies make use of a germline-encoded Trp residue to bind to the turns and extended conformations of the NANP repeats. In addition, we observe a recurring structural pattern of how these repeats are linked together when complexed with protective antibodies (type I β-turn and Asn pseudo 3_10,_ as in mode 1) that relates to the repeating unit in the long-range spiral structure found in the complex of Fab311 with rsCSP [34]. As our understanding of the antibody structural features that can be correlated with *bona fide* protection for *P. falciparum* continues to be expanded and refined, these different binding modes of antibody to the NANP repeats can be harnessed in vaccine design to selectively elicit a wide range of protective antibodies.

## Materials and Methods

### Mouse liver burden assay and mosquito bite challenge

Female, 6–8 weeks old C57Bl/6 mice were purchased from Charles River. To measure the liver burden, mice (*N* = 5) were IV injected with 100 μg of Ab per mouse and, 16 h later, challenged IV with 2000 *P. berghei* transgenic sporozoites expressing the *P. falciparum* CSP and luciferase. 42 h after challenge, mice were injected IP with 100 μl of D-luciferin (30 mg/mL), having been anesthetized by exposure to isoflurane. Bioluminescence in the liver was measured using an IVIS Spectrum (Perkin Elmer, Waltham, MA). Regarding sterile protection, mice (*N* = 6) were passively immunized with 300 μg of Ab and, 16 h later, were challenged by a 10-min exposure to the bites of six mosquitoes of which five are on average infected with the transgenic parasite. Parasite infection of red blood cells was assessed from day 4 after challenge by microscopic observation of blood smears. All procedures were performed according to ACUC procedures at Johns Hopkins University.

### Sporozoite enzyme-linked immunosorbent assay

*P. falciparum* sporozoites (5000, suspended in 100 μl of Hanks Balanced Saline solution) were placed in ELISA wells (Nunc Maxisorp™; Thermo Fisher Scientific, Waltham, MA) and subjected three times to freeze and thaw. For the assay, the wells were washed and incubated for 1 h with PBS containing 1% BSA. Different Abs at different concentrations were then placed in the wells at an initial concentration of 1 μg/ml followed by serial threefold dilutions. After 1 h, plates were washed and incubated with a secondary antibody, HRP-conjugated antihuman IgG (Jackson Immunoresearch, West Grove, PA), washed after 1 h, and then developed with ABTS substrate. The experiment was conducted in duplicate (*N* = 2).

### Protein production

All of the Fabs used in this study were expressed in Chinese hamster ovary cells (ExpiCHO; Thermo Fisher Scientific, Waltham, MA) and purified using a HiTrap Protein G HP column (GE Healthcare, Chicago, IL) followed by size-exclusion chromatography (Superdex 200 16/90; GE Healthcare, Chicago, IL) in Tris Buffered Saline (TBS: 50 mM Tris pH 8.0, 137 mM NaCl, 3.6 mM KCl). Wild-type IgG1 397, 311, and 317 for the protection study were expressed in HEK293F cells (Thermo Fisher Scientific, Waltham, MA), purified using HiTrap Protein G HP column (GE Healthcare, Chicago, IL), and washed with 0.5 M Arginine in Dulbecco's phosphate-buffered saline pH 7.3 as described previously [49] to remove possible endotoxin contamination. Endotoxin level was checked using Endosafe® nexgen-PTS™ portable endotoxin testing system (Charles River, Wilmington, MA). rsCSP was expressed in *E. coli* (SHUFFLE cells; New England Biolabs, Ipswich, MA) and purified as described [29].

### Crystallization and structural analysis

Six substitutions and one deletion (from ^112^SSASTKG^118^ to ^112^VSRRLP^117^) were introduced into the elbow region of Fab397 heavy chain to stabilize the Fab and facilitate crystallization as previously described [39]. Fab397 was concentrated to 10 mg/ml and mixed with a 5:1 M ratio of NPNA_4_ peptide to Fab. Crystal screening was carried out using our high-throughput, robotic CrystalMation system (Rigaku, Carlsbad, CA) at The Scripps Research Institute, which was based on the sitting drop vapor diffusion method with 35 μL reservoir solution and each drop consisting 0.1 μL protein +0.1 μL precipitant. High-quality crystals that diffracted to high resolution (1.75 Å) were obtained using a reservoir solution containing 0.1 M HEPES pH 6.62 and 22% (w/v) PEG 4000. Crystals were grown at 20 °C and appeared after 7 days. Fab397-peptide crystals were cryoprotected by soaking in a well solution supplemented with 20% PEG200 before being flash-cooled in liquid nitrogen. X-ray diffraction data were collected at the Advanced Proton Source (APS) beamline 23ID-B. The dataset was indexed, integrated, and scaled using the HKL-2000 package [50]. The structure was determined by molecular replacement using Phaser [51] and a homology model generated from PIGSPro [52] as a search model. Structure refinement was performed using phenix.refine [53] and iterations of refinement using Coot [54]. Amino-acid residues of the Fabs were numbered using the Kabat system. The structure was validated using MolProbity [55].

Buried surface areas (BSAs) were calculated with the program MS [56]. Calculation of dihedral angles was performed using ANGLES (CCP4) [57]. Hydrogen bonds were assessed with the program HBPLUS [58]. Ramachandran plots for the NPNA-repeat peptides were constructed in R with the previously reported φ, ψ angle distribution [59]. The composite omit map was calculated using Composite Omit Map tool in Phenix with the “simple” method as described [60]. CH–π interactions were identified based on the criteria described in previous study [61] (see also Table 3), whereas π–π interactions were characterized based on parameters previously reported [62].

### Isothermal titration calorimetry (ITC)

Titrations were performed on a MicroCal Auto-iTC200 (GE Healthcare, Chicago, IL). Prior to the experiments, wild-type Fab397 was extensively dialyzed against Dulbecco's PBS (Thermo Fisher, Waltham, MA). The peptides were placed in the syringe at a concentration of 157 μM for Ac-NPNANPNA-NH2, 104 μM for AcNPNANPNANPNANPNA-NH2, and 48 μM for Ac-NPNANPNANPNANPNA NPNANPNANPNANPNA -NH2, whereas the concentration of Fab in the cell was 8.9 μM for all experiments. The Fab and peptide concentrations were determined by UV absorbance at 280 nm and 205 nm. Molar extinction coefficients for the peptides at 205 nm were estimated using a previously published method [63]. The titrations were all performed with peptides in the syringe and antibodies in the cell and consisted of 16 injections of 2.45 μL peptide at a rate of 0.5 μl/s at 120 s time intervals, with injection duration of 4.9 s, injection interval of 180 s, and reference power of 5 μCal. Experiments were conducted in quadruplet (*N* = 4) at 25 °C. Fitting of the integrated titration peaks was performed with Origin 7.0 software using a single-site binding model. The first data point was excluded from the fit as commonly done.

### Biolayer interferometry

NANP binding of Fab397, Fab311, and Fab317 wild-type and mutants was determined using biolayer interferometry (Octet Red; Pall ForteBio, Fremont, CA). Biotinylated peptides, Biotin-linker-NPNANPNANPNA-NH_2_ (NPNA)_3_, were ordered from Innopep Inc. The peptides were loaded onto streptavidin biosensors (Pall ForteBio, cat No 18-5019) at 10 μg/mL in kinetics buffer (Dulbecco's PBS + 0.002% Tween20 and 0.01% BSA). The loaded sensors were dipped into solutions containing dilutions of each Fab in kinetics buffer at a concentration of 2000 nM. The binding experiments were performed with the following steps: 1) baseline in kinetics buffer for 60 s, 2) loading of peptide for 120 s, 3) baseline for 60 s, 4) association of antibody for 120 s, and 5) dissociation of antibody into kinetics buffer for 150 s. A reference well with no peptide loaded onto the biosensor was run in all experiments and subtracted from sample wells to correct for drift and buffer evaporation. Octet assays were carried out at room temperature. Data were analyzed using the Octet Red Data Analysis software version 9.0.

### Negative-stain electron microscopy

Samples were diluted to 0.01 mg/ml in 1× TBS, pH 7.4 and 3 μL were applied to a copper mesh grid (Electron Microscopy Sciences, Hatfield, PA) for 5 s, respectively. The grids had been recently plasma cleaned (Gatan, Inc., Pleasanton, CA) for 20 s using an Argon/Oxygen mix. 2% uranyl formate was used to stain the grids for 50 s. The nsEM data were collected on a Thermo Fisher Tecnai Spirit (120 kV) with a Tietz 4Kx4k camera and automated using the Leginon software [64]; all images were stored in the Appion database [65]. Particles were picked using DogPicker [66] and stacked with a box size of 160 or 192 pixels for Fab397-NPNA_8_ and Fab397-rsCSP, respectively. CTF estimation was performed with GCTF [67] and particles were extracted with a box size of 160 or 192 pixels. The particle stacks were imported into cryoSparc2 [68] for 2D classification, 3D classification, and final 3D refinements. Final reconstructions were evaluated in UCSF Chimera [69].

### Statistical analysis

Data for sporozoite ELISA assay (*N* = 2) were fitted and plotted using GraphPad Prism, version 7.0. For mouse liver burden assay (*N* = 5 mice), data were compared for significance using a Mann–Whitney *U* test with *p* < 0.05 indicated a statistically significant difference. Data were reported as the geometric mean of the total flux with 95% confidence interval (Fig. 1c) and as the arithmetic mean of % inhibition with error bars indicating two standard deviations (2SD; Fig. 1d). All statistical parameters and graphs for the mouse liver burden assay were calculated and plotted using R with the Hmisc and ggplot2 packages. Each ITC experiment was performed with four replicates (*N* = 4), and the data were reported as the arithmetic mean ± SD.

## Accession Numbers

The crystal structure of Fab397 in complex with the NPNA_4_ peptide has been deposited in the Protein Data Bank with accession code 6UC5. The antibody structures used for comparison of the Trp residues and binding to the type I β-turn were obtained from the Protein Data Bank: Fab311 and Fab317 (PDB ID: 6AXK and 6AXL, respectively) [29]; Fab663 (PDB ID: 5BK0) [33]; Fab1210 and 1450 (PDB ID: 6D01 and 6D11, respectively) [31]; FabCIS43 (PDB ID: 6B5O) [30]; and FabMGG4 (PDB ID: 6BQB) [32]. The crystal structure of the NANPNA peptide was obtained from the publication site [35]. The EM reconstructions and maps are deposited in the Electron Microscopy Data Bank: Fab397-NPNA_8_ class 1–3 (EMDB ID: EMD-20772, EMD-20773, and EMD-20774, respectively) and Fab397-rsCSP class 1–6 (EMDB ID: EMD-20775, EMD-20776, EMD-20777, EMD-20778, EMD-20779, and EMD-20780, respectively).
