# Supplementary material for: Diverse Antibody Responses to Conserved Structural Motifs in Plasmodium falciparum Circumsporozoite Protein
Source: J Mol Biol. 2020 Feb 14;432(4):1048–63. doi: 10.1016/j.jmb.2019.12.029 (PMC7057269; doi:10.1016/j.jmb.2019.12.029)
Supplement: Multimedia component 1 [file mmc1.docx]

**Supplementary information**

**Diverse antibody responses to conserved**

**structural motifs in *Plasmodium falciparum***

**circumsporozoite protein**

**Tossapol Pholcharee^a^, David Oyen^a^, Jonathan L. Torres^a^, Yevel Flores-Garcia^b^, Gregory M. Martin^a^, Gonzalo Gonzalez-Paez^a^, Daniel Emerling^c^, Wayne Volkmuth^c^, Emily Locke^d^, C. Richter King^d^, Fidel Zavala^b^, Andrew B. Ward^a^, Ian A. Wilson^a,e^**

^a^Department of Integrative Structural and Computational Biology, The Scripps Research Institute, La Jolla, CA 92037, USA.

^b^Malaria Research Institute, Johns Hopkins Bloomberg School of Public Health, Baltimore, MD 21204, USA.

^c^Atreca Inc., Redwood City, CA 94063 , USA.

^d^PATH’s Malaria Vaccine Initiative, PATH Center for Vaccine Innovation and Access, Washington, DC 20001, USA.

^e^The Skaggs Institute for Chemical Biology, The Scripps Research Institute, La Jolla, CA 92037, USA.

Corresponding author:

Ian Wilson

The Scripps Research Institute, 10650 North Torrey Pines Road, MailBox: BCC206, La Jolla, California, United States 92037. Tel: +1 858-784-9706; Fax: +1 858-784-2980; Email: wilson@scripps.edu
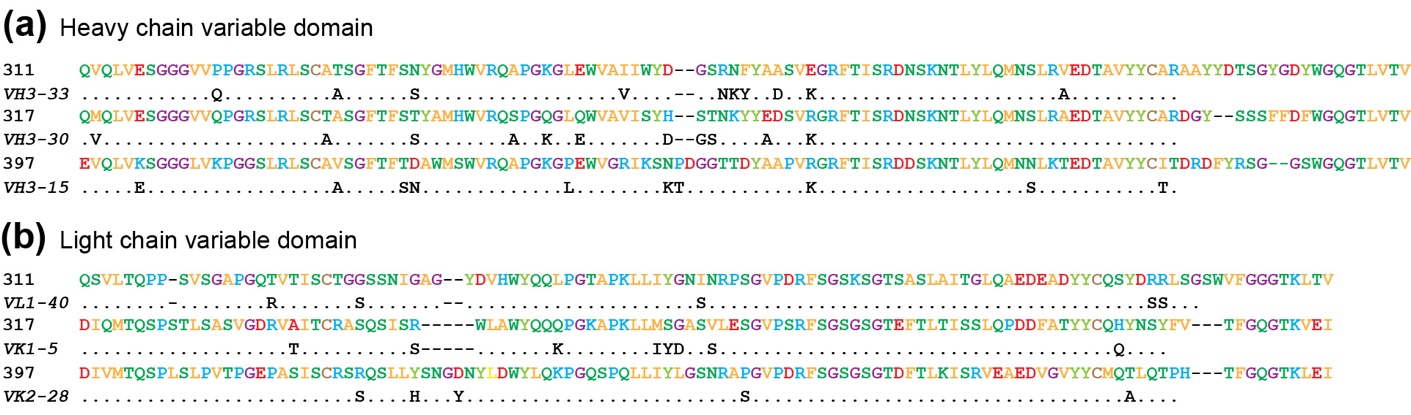


**Fig. S1.** Sequence alignment of certain antibodies obtained from the MAL071 RTS,S clinical trial from different germline genes. The characterization of mAb311 and mAb317 have been previously reported [1, 2]. The protein sequences include (a) the heavy chain variable domain and (b) the light chain variable domain. The germline sequence is provided below each antibody sequence. The dots in the germline sequences indicate the same residues as the antibody sequence, and only different residues are clearly shown.

_
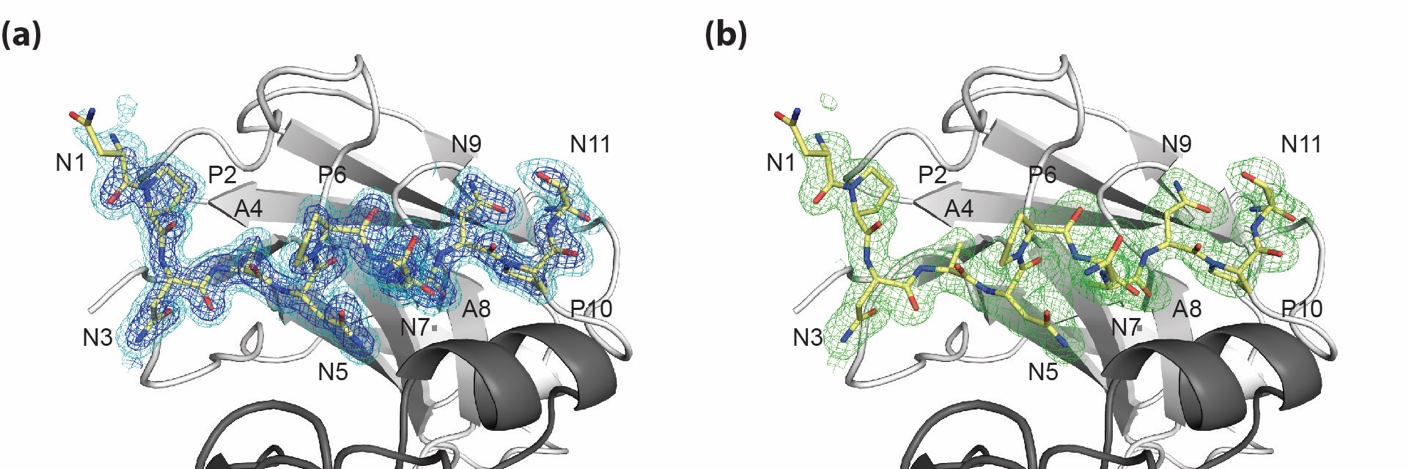
_

**Fig. S2.** Electron density of Fab397-bound peptide. (a) 2Fo-Fc (blue) and (b) composite omit 2Fo-Fc (green) electron density map for the peptide (yellow carbons) bound to Fab397. The 2Fo-Fc density is contoured at 2.0σ (dark blue) and 0.8σ (cyan), while the omit 2Fo-Fc density is contoured at 0.8σ. The Fab binding site is shown in cartoon representation with the heavy and light chains colored charcoal and light grey, respectively. Residues (1-11) of the NPNA_4_ peptide that are observed in the electron density map are labeled.


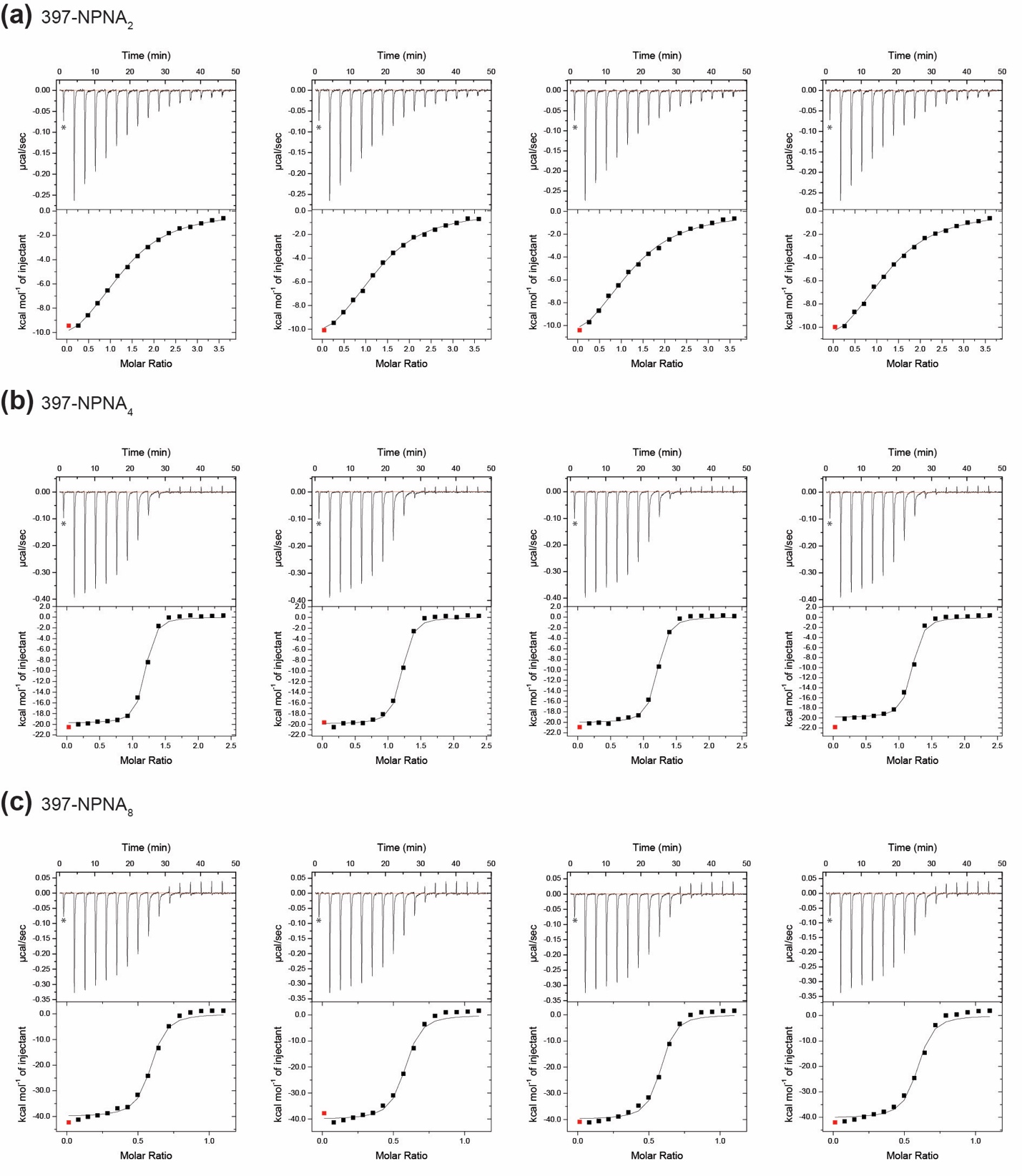


**Fig. S3.** ITC binding curves for Fab397 binding to NANP repeat peptides. ITC binding data for Fab397 with: (a) 8-mer peptide Ac-NPNANPNA-NH2, (b) 16-mer peptide Ac-NPNANPNA NPNANPNA-NH2, and (c) 32-mer Ac-NPNANPNA NPNANPNA NPNANPNA NPNANPNA-NH2. Data points not included in the fit are indicated by an asterisk.

**
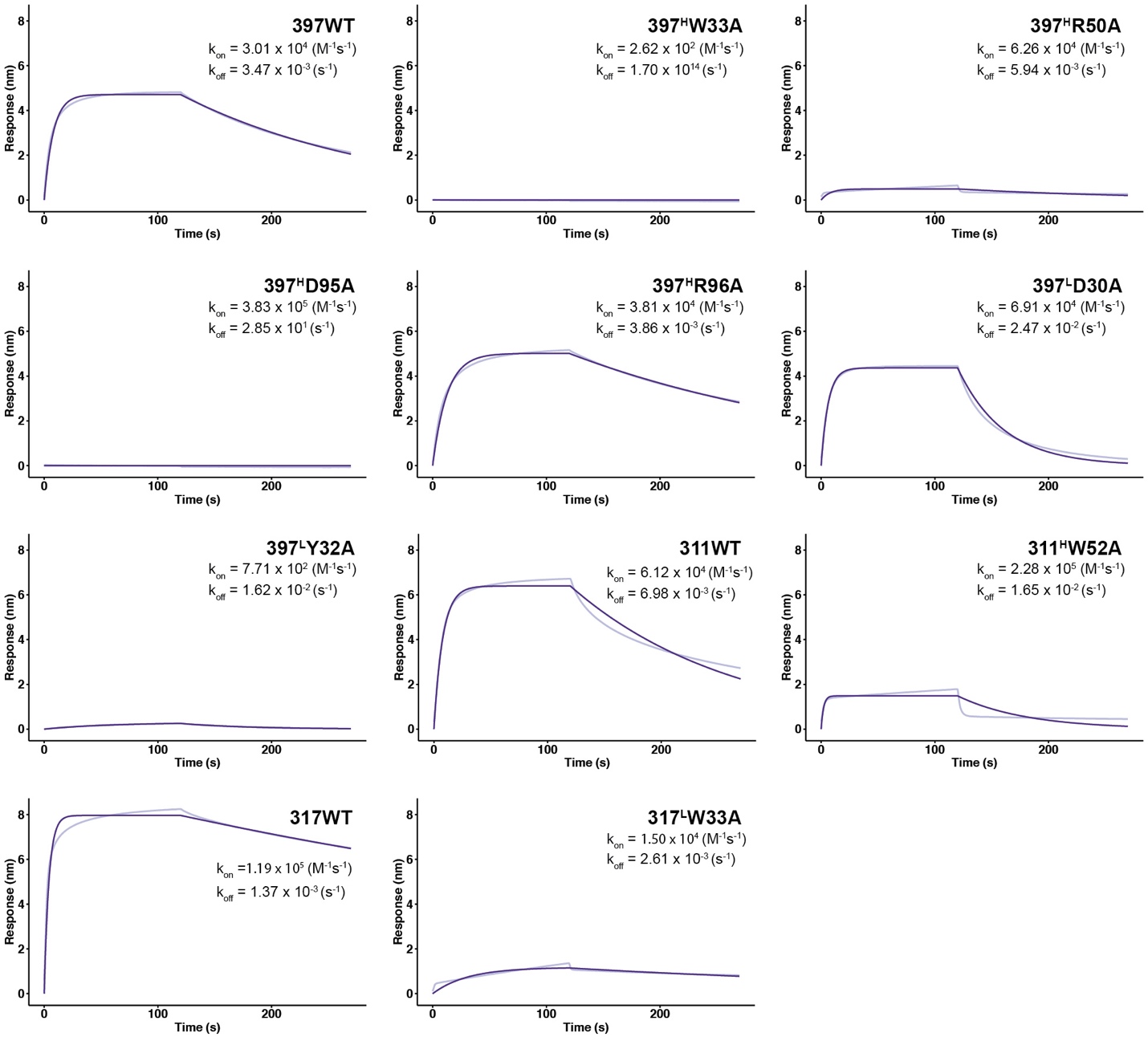
**

**Fig. S4.** Kinetics of binding for Fab397, Fab311, Fab317, and their mutants to the NPNA_3_ peptide. Binding was assessed using bio-layer interferometry (BLI). Binding curves are shown in light purple and fits are shown in dark purple. The rate constants for each Fab estimated from the fits are also reported.


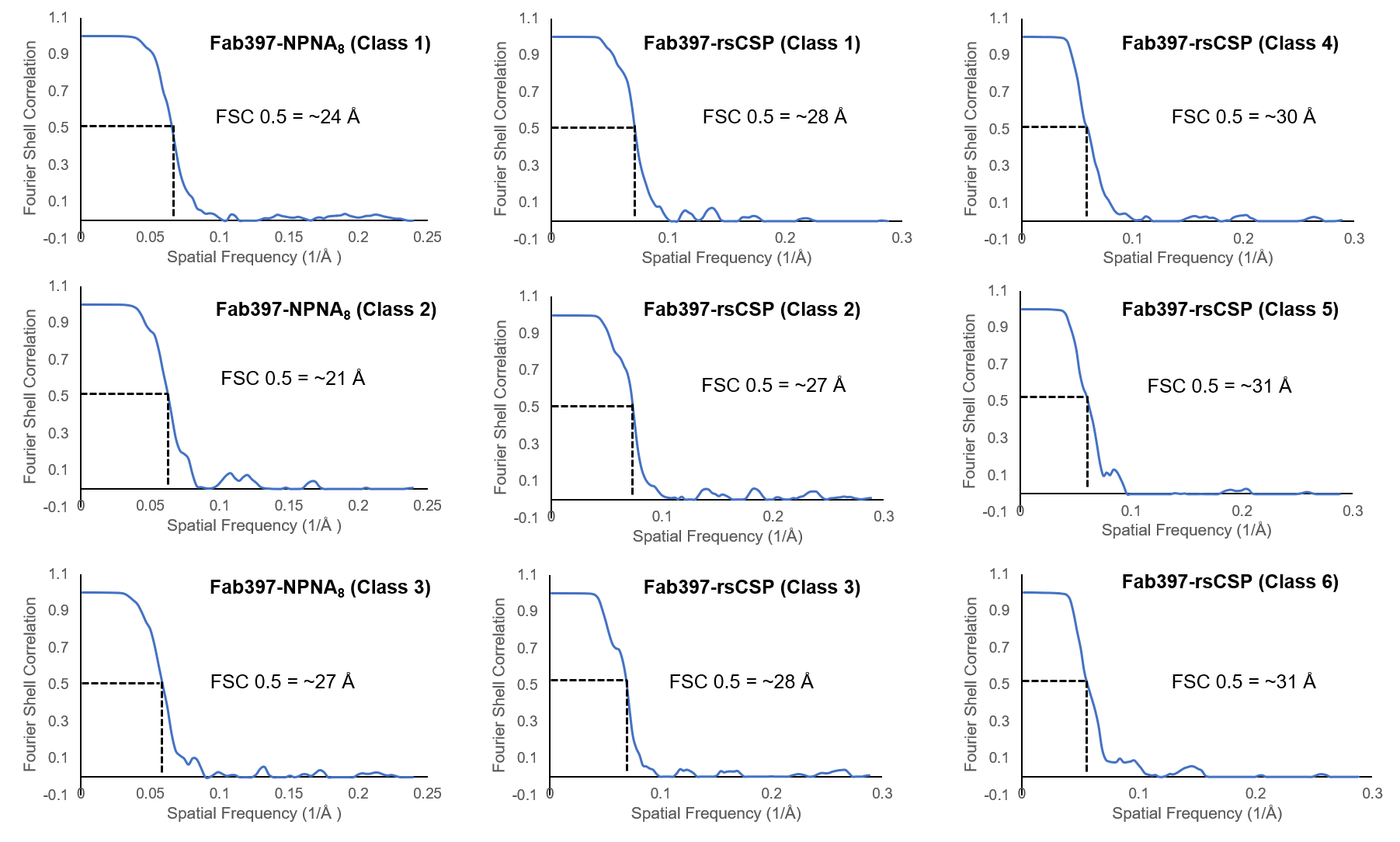


**Fig. S5.** Fourier shell correlation curves for the Fab397-NPNA_8_ and Fab397-rsCSP classes from nsEM. Calculated resolution for all nsEM maps is around 21-31 Å.

**
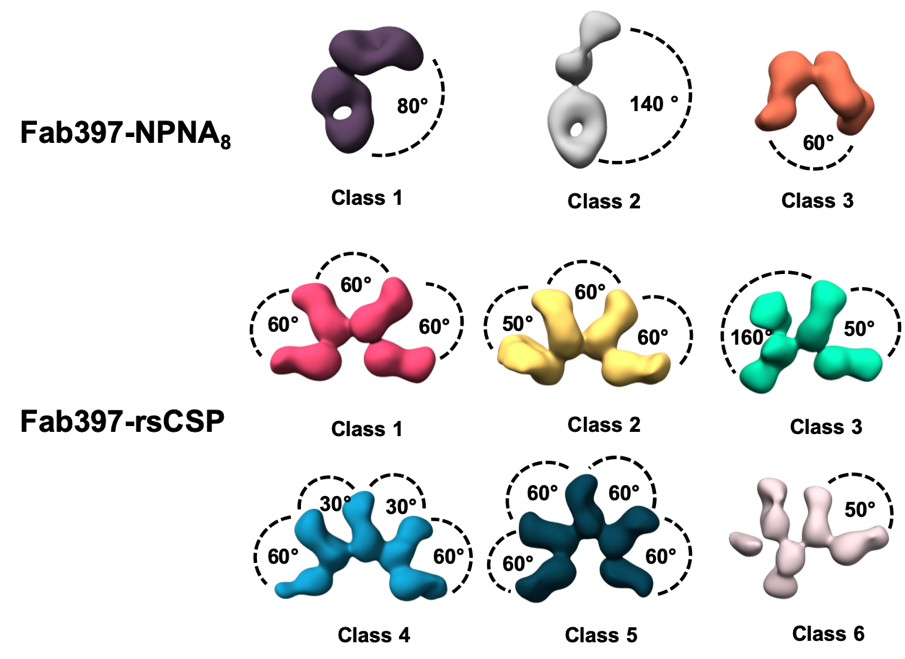
**

**Fig. S6.** Different Fab397 arrangements in NPNA_8_ and rsCSP complexes. The crystal structure of Fab397 was docked into the refined nsEM classes of Fab397-NPNA_8_ or Fab397-rsCSP complexes. Relative Fab angles were estimated in UCSF Chimera by selecting 3 atoms: a central atom was placed where the Fabs intersect and one atom was picked in the center of two different Fabs.

**
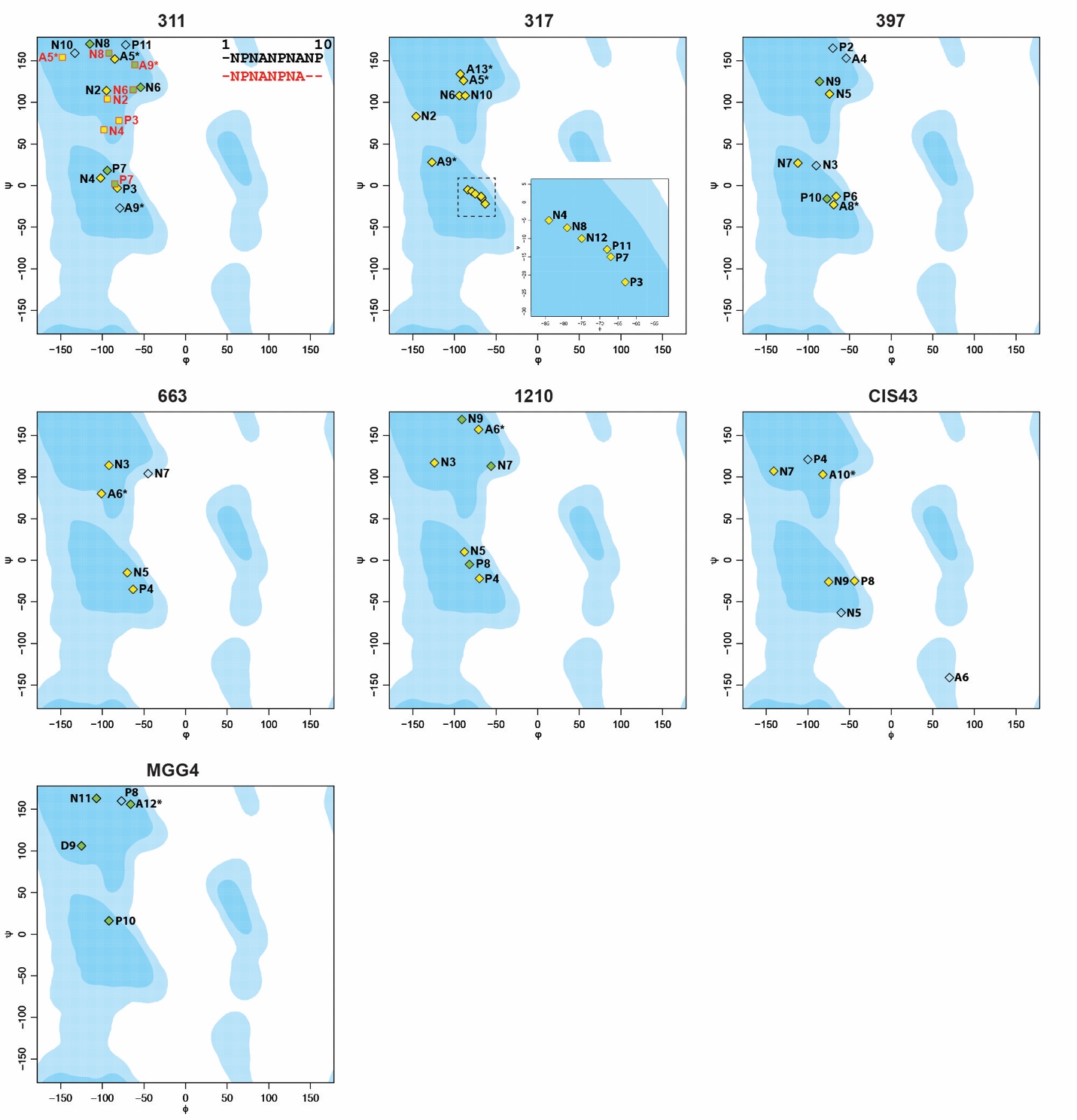
**

**Fig. S7.** Ramachandran plots of dihedral angles from anti-CSP-antibody-bound peptides that contain a type I β-turn or Asn pseudo 3_10_ turn. Dihedral angles of the NPNA motif involved in type I β-turns and Asn pseudo 3_10_ turns are shown as yellow and green diamonds, repectively. The terminal Ala residues from both turns are indicated with asterisks. For Fab311, dihedral angles from the repeat peptides found in the crystal structure [1] (black diamonds with black labels) and from rsCSP in the Fab311-rsCSP cryo-EM structure [2] (red bordered square with red atom labels) are plotted, and the sequence alignment is shown (peptide from crystal structure = black, repeating NPNANPNA motif from rsCSP = red). For the NPNANPNA motif from the Fab311-rsCSP structure, the average values of dihedral angles of four motifs are plotted (see Table S3). For Fab317-bound peptide plot, the area enclosed by dashed square is enlarged on the bottom right of the plot for clarity.

**Table S1.** Evaluation of protective capacity of mAb311 and mAb397 in mice.

| Cohort | Mouse | Mosquitoes positive for bloodmeal | Blood-stage parasitemia | | | | | | | Infected/  challenged (mice) | Pre-patent period (days) | Protection |
| --- | --- | --- | --- | --- | --- | --- | --- | --- | --- | --- | --- | --- |
|  |  |  | D4 | D5 | D6 | D7 | D8 | D10 | D12 |  |  |  |
| Naïve | 1 | 5/6 | + |  |  |  |  |  |  | 6/6 | 4 | 0% |
|  | 2 | 5/6 | + |  |  |  |  |  |  |  |  |  |
|  | 3 | 5/6 | + |  |  |  |  |  |  |  |  |  |
|  | 4 | 6/6 | + |  |  |  |  |  |  |  |  |  |
|  | 5 | 5/6 | + |  |  |  |  |  |  |  |  |  |
|  | 6 | 6/6 | + |  |  |  |  |  |  |  |  |  |
| mAb311  300μg/  mouse | 1 | 6/6 | - | - | - | - | - | - | - | 0/6 | N/A | 100% |
|  | 2 | 6/6 | - | - | - | - | - | - | - |  |  |  |
|  | 3 | 4/6 | - | - | - | - | - | - | - |  |  |  |
|  | 4 | 4/6 | - | - | - | - | - | - | - |  |  |  |
|  | 5 | 5/6 | - | - | - | - | - | - | - |  |  |  |
|  | 6 | 6/6 | - | - | - | - | - | - | - |  |  |  |
| mAb397  300 μg/  mouse | 1 | 6/6 | - | - | - | - | - | - | - | 0/6 | N/A | 100% |
|  | 2 | 6/6 | - | - | - | - | - | - | - |  |  |  |
|  | 3 | 5/6 | - | - | - | - | - | - | - |  |  |  |
|  | 4 | 5/6 | - | - | - | - | - | - | - |  |  |  |
|  | 5 | 6/6 | - | - | - | - | - | - | - |  |  |  |
|  | 6 | 5/6 | - | - | - | - | - | - | - |  |  |  |

Mice were infected by mosquito bite challenge with *P. berghei* chimeric parasites expressing full-length PfCSP. Note: Grey boxes indicate the mice that were diagnosed as parasite-infected and euthanized following ACUC recommendations.

**Table S2.** Hydrogen bonds between Fab397 and (NPNA)_4_ peptide with BSA.

| NPNA_4_ (BSA Å^2^) | Fab397 residues |  | Distance (Å) |
| --- | --- | --- | --- |
| Asn3 (70) |  |  |  |
| Asn-O | ^H^Arg^50^-NH1, ^H^Arg^50^-NH2 |  | 3.22, 3.20 |
| Asn-N | ^L^Gln^93^-OE1 |  | 3.07 |
| Asn-OD1 | ^L^Thr^94^-N |  | 3.13 |
| Asn-ND2 | ^L^Thr^94^ -OG1 |  | 2.76 |
| Asn5 (73) |  |  |  |
| Asn-ND2 | ^H^Asp^95^-OD2 |  | 3.07 |
| Asn-O | ^L^Tyr^32^-OH |  | 2.62 |
| Asn7 (32) |  |  |  |
| Asn-O | ^H^Arg^96^-NH1 |  | 2.80 |
| Asn9 (47) |  |  |  |
| Asn-ND2 | ^L^Asp^30^-OD2 |  | 2.83 |
| Asn11 (24) |  |  |  |
| Asn-ND2 | ^L^Tyr^49^-OH |  | 2.99 |

**Table S3.** Dihedral angles of NPNA residues that form type I β-turns and Asn pseudo 3_10_ turns in NANP repeat peptides. The NPNA conformations are from crystal and cryo-EM structures with anti-CSP antibodies and from the NANPNA crystal structure reported in Ghasparian *et al*., 2006 [3]. These turns are classified here into different modes based on the Ψ angle of the terminal Ala residue and are colored respectively.

| **Antibody** | **φ (**º**)** |  |  |  | **Ψ(**º**)** |  |  |  |  |
| --- | --- | --- | --- | --- | --- | --- | --- | --- | --- |
|  | **N** | **P** | **N** | **A** | **N** | **P** | **N** | **A** | **Mode** |
| **Type I β-turn** | **i** | -60 | -90 | **i+3** | **i** | -30 | 0 | **i+3** |  |
| 311 | -95 | -82 | -102 | -85 | 114 | -3 | 9 | 152 | 1 |
| 317-1 | -146 | -63 | -84 | -89 | 83 | -22 | -5 | 126 | 1 |
| 317-3 | -87 | -68 | -75 | -93 | 108 | -13 | -10 | 134 | 1 |
| 1210 | -124 | -70 | -88 | -71 | 117 | -22 | 10 | 157 | 1 |
| 663 | -92 | -63 | -70 | -101 | 114 | -35 | -15 | 80 | 1 |
| CIS43 | -141 | -44 | -75 | -82 | 107 | -25 | -26 | 103 | 1 |
| **Average** | -114 | -65 | -82 | -87 | 107 | -20 | -6 | 125 | 1 |
| **SD** | 26 | 12 | 12 | 10 | 12 | 11 | 14 | 29 | 1 |
| 317-2 | -94 | -67 | -79 | -127 | 108 | -15 | -7 | 28 | 2 |
| 397 | -74 | -66 | -112 | -69 | 110 | -13 | 27 | -23 | 3 |
| Ghasparian  et al., 2006 | -69 | -71 | -110 | -81 | 118 | -6 | 16 | 165 | 1 |
| **Pseudo 3_10_** |  |  |  |  |  |  |  |  |  |
| 311(rsCSP)* | -63 | -85 | -92 | -61 | 115 | 2 | 159 | 145 | 1 |
| MGG4 | -125 (D)^#^ | -92 | -107 | -66 | 106 (D) | 16 | 163 | 156 | 1 |
| 1210 | -56 | -82 | -91 | -64 | 113 | -5 | 169 | N/A | N/A |
| 397 | -86 | -77 | -92 | N/A | 125 | -16 | N/A | N/A | N/A |

*The spiral rsCSP cryoEM structure has 11 NPNANPNA epitopes for Fab 311 [2]. The terminal epitopes were excluded as well as non-NANP epitopes. Dihedral angles of the four NPNANPNA epitopes (^135^N to A^166^) were averaged and reported here.

^#^ D is present instead of N in the peptide in the MGG4 complex

N/A means that the dihedral angles are not available for that particular residue because: (1) the residue is not present in the crystal structure; or (2) the amino acid is the final residue in the peptide chain.

**References**

[1] D. Oyen, J. L. Torres, U. Wille-Reece, C. F. Ockenhouse, D. Emerling, J. Glanville, et al. Structural basis for antibody recognition of the NANP repeats in *Plasmodium falciparum* circumsporozoite protein. Proc Natl Acad Sci U S A. 2017;114:E10438-E45.

[2] D. Oyen, J. L. Torres, C. A. Cottrell, C. R. King, I. A. Wilson, A. B. Ward. Cryo-EM structure of *P. falciparum* circumsporozoite protein with a vaccine-elicited antibody is stabilized by somatically mutated inter-Fab contacts. Sci Adv. 2018;4:eaau8529.

[3] A. Ghasparian, K. Moehle, A. Linden, J. A. Robinson. Crystal structure of an NPNA-repeat motif from the circumsporozoite protein of the malaria parasite *Plasmodium falciparum*. Chem Commun (Camb). 2006;2:174-6.
